# W-Band Low-Noise Amplifier with Improved Stability Using Dual RC Traps in Bias Networks on a 0.1 μm GaAs pHEMT Process

**DOI:** 10.3390/mi16020219

**Published:** 2025-02-15

**Authors:** Seong-Hee Han, Dong-Wook Kim

**Affiliations:** Department of Radio and Information Communications Engineering, Chungnam National University, 99 Daehak-ro, Yuseong-gu, Daejeon 34134, Republic of Korea; hee.hans@o.cnu.ac.kr

**Keywords:** GaAs pHEMT, low-noise amplifier, MMIC, network determinant function, RC trap

## Abstract

This paper demonstrates that potential oscillations in various frequency bands of monolithic microwave integrated circuits (MMICs) can be effectively suppressed using well-designed dual RC traps in the bias networks. The proposed approach is applied to the design and development of a highly stable W-band low-noise amplifier (LNA) MMIC for high-precision millimeter-wave applications. The amplifier is fabricated using the 0.1 µm GaAs pHEMT process from Win Semiconductors. The cascaded four-stage design consists of two low-noise-optimized stages, followed by two high-gain-tuned stages. Stability is enhanced through the integration of dual RC traps in the bias networks, which is rigorously evaluated using stability factors (K and μ) and network determinant function (NDF) encirclement analysis. In low-noise mode, the developed low-noise amplifier MMIC achieves a noise figure of 5.6−6.2 dB and a linear gain of 17.8−19.8 dB over the 90−98 GHz frequency range, while only consuming a DC power of 96 mW. In high-gain mode, it has a noise figure of 6.2−6.9 dB and a linear gain of 19.8−21.7 dB.

## 1. Introduction

The W-band frequency range has become crucial for enabling high data rates and a wide bandwidth in wireless communications and remote sensing applications [1,2,3,4]. In these systems, low-noise amplifiers (LNAs) are essential for effective signal reception, preserving signal integrity and maintaining an optimal signal-to-noise ratio (SNR). LNAs in millimeter-wave systems require the careful optimization of noise source impedance to minimize a noise figure, while ensuring sufficient gain to sustain the overall system SNR.

Various semiconductor technologies have been used in the development of W-band LNAs, including GaAs pseudomorphic high-electron-mobility transistors (pHEMTs), GaAs metamorphic high-electron-mobility transistors (mHEMTs), Si complementary metal oxide semiconductor (CMOS) field-effect transistors, GaN HEMTs, and InP HEMTs [5,6,7]. These device technologies offer diverse trade-offs in performance, with each technology demonstrating specific advantages for different operational conditions and application requirements. Si CMOS technology has advantages such as low power consumption, low production cost, and easy integration with digital devices, but its low f_T_ and f_max_ limit its performance and utilization in W-band applications [8,9]. InP HEMTs and mHEMTs can achieve the highest f_T_ and f_max_ due to their superior material properties, providing sufficient gain and a low noise figure, but their low breakdown voltage limits their power-handling capabilities, and their high processing cost makes mass production challenging [10,11,12]. GaN HEMTs have a high breakdown voltage and are well suited for high-power applications, but their noise performance is inferior to that of InP HEMTs and mHEMTs, which makes GaN HEMTs less suitable for low-noise applications [13,14,15]. While they have a slightly lower gain and worse noise performance compared to InP HEMTs and mHEMTs, GaAs pHEMTs benefit from a well-established fabrication process and offer a good balance between high gain and low noise in the W-band [16,17,18].

W-band amplifiers are highly susceptible to power supply noise, the parasitic effects of circuit elements, and unwanted reflections from discontinuities, which can degrade the amplifier’s stability. The stability of W-band amplifiers can be achieved by carefully considering the stability criteria and circuit element tuning during design. Typically, approaches to enhancing circuit stability include performing stability analysis under both small-signal and large-signal operation conditions and applying techniques such as low-pass or high-pass filtering, lossy networks, negative feedback, and stable grounding using high-value bypass capacitors, in impedance matching and bias networks [19,20,21,22].

In this paper, the inherent stability issues of GaAs pHEMTs are addressed, and the effective suppression of potential oscillations in monolithic microwave integrated circuits (MMICs) is demonstrated using well-designed dual RC traps in bias networks. A circuit approach for stable operation is proposed and applied to the design of a four-stage low-noise amplifier optimized for operation at 94 GHz. The stabilization approach is rigorously evaluated using network determinant function (NDF) analysis to ensure reliable performance under various conditions. The four-stage amplifier, fabricated using a 0.1 µm GaAs pHEMT process [23,24,25], operates in dual modes to deliver both high gain and low noise, and its measured results are presented.

## 2. Circuit Design

### 2.1. Device Selection

**Figure 1:**
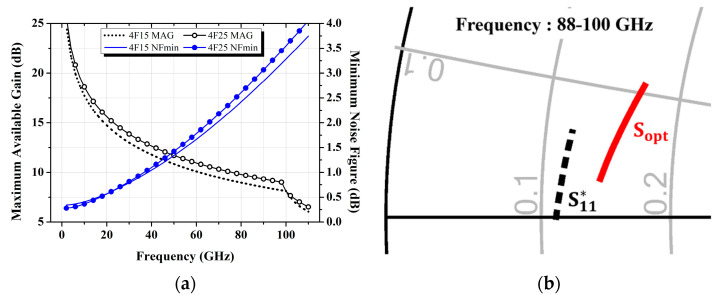
Frequency responses and target impedance traces of the 4 × 15 μm and 4 × 25 μm transistors under the bias conditions (V_GS_ = −0.4 V and V_DS_ = 2 V): (**a**) Maximum available gain and minimum noise figure; (**b**) S_11_^*^ and S_opt_.

The selection of active devices is driven by the specific requirements of the gain and noise figure in amplifier design. In this study, a four-stage low-noise amplifier is designed using two 4 × 15 μm and two 4 × 25 μm pHEMTs, fabricated using a 0.1 μm pHEMT process from Win Semiconductors (Tao Yuan, Taiwan). The first and second stages are optimized to achieve the minimum noise figure, while the third and fourth stages are designed for optimal gain through conjugate impedance matching. Furthermore, the unit gate width, the number of gate fingers and the bias conditions for each transistor are carefully chosen to achieve the lowest obtainable noise figure. The maximum available gain (MAG) and minimum noise figure (NF_min_) characteristics of both 4 × 15 μm and 4 × 25 μm transistors are depicted in Figure 1a, and the conjugate input reflection coefficient (S_11_*) and the optimum source impedance (S_opt_) for the 4 × 15 μm transistor are shown as an example in Figure 1b.

### 2.2. Stabilization with Dual RC Traps

**Figure 2:**
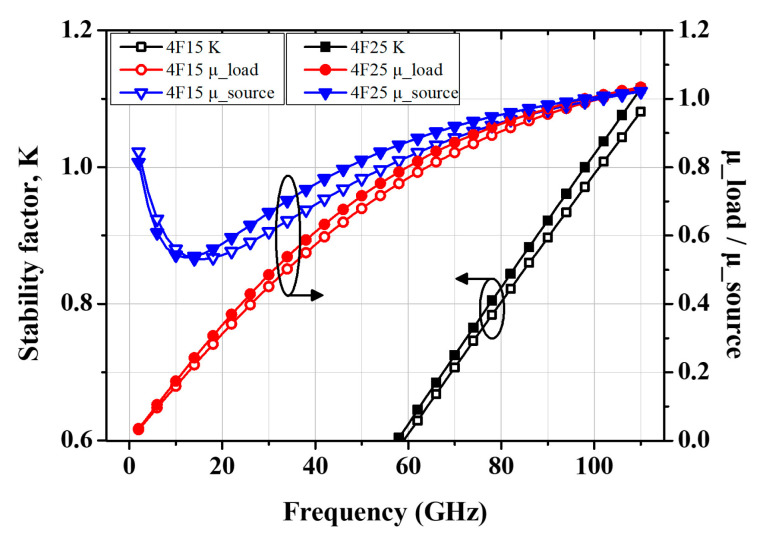
Stability factors K and μ (at source and load) of the 4 × 15 μm and 4 × 25 μm transistors under the class AB bias conditions (V_GS_ = −0.4 V and V_DS_ = 2 V).

Figure 2 shows the simulation results of the stability factors, K and μ, for the 4 × 15 µm and 4 × 25 µm pHEMTs under class AB bias conditions (V_GS_ = −0.4 V and V_DS_ = 2 V). Stability factors below 1 within the low- and design-frequency ranges may lead to amplifier instability, necessitating stabilization steps to mitigate oscillations [26].

**Figure 3:**
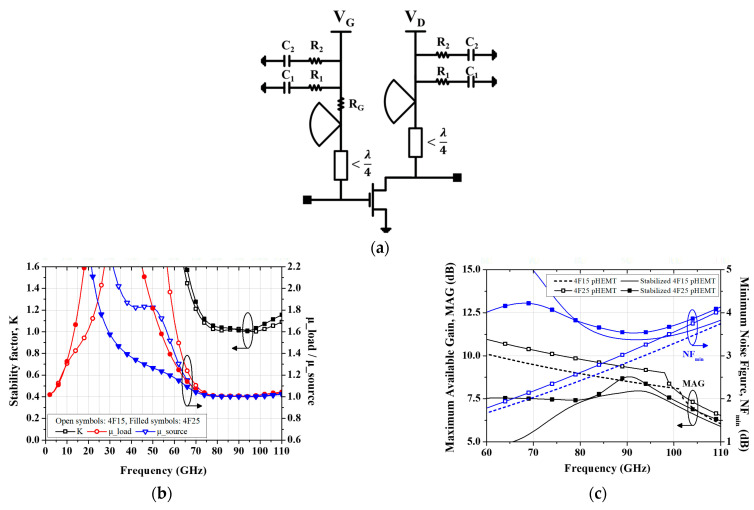
Stabilized unit transistor circuit and its results: (**a**) Stabilization circuit with dual RC traps (R_1_ = 40 Ω, C_1_ = 0.2 pF, R_2_ = 20 Ω, C_2_ = 0.9 pF); (**b**) Stability factors; (**c**) Maximum available gain (black lines) and minimum noise figure (blue lines).

Figure 3a shows a schematic circuit diagram with the stabilization techniques implemented in the bias networks. Radial stubs with an electrical length of λ/4 are employed to make virtual ground nodes at the design frequency, effectively preventing RF signals from propagating into the bias supply ports [27,28,29]. In addition, in the gate bias networks, resistors (R_G_) may be placed in series with shunt bypass capacitors or shunt bypass RC traps, thereby suppressing loop feedback oscillations through the bias supply ports at low frequencies [30,31]. Figure 3b,c show the effects of the stabilization circuits on the stability factors, maximum available gain and minimum noise figure. The results demonstrate that all stability factors exceed 1 across the entire frequency range. While the maximum available gain decreases by approximately 0.4 dB, and the minimum noise figure degrades by about 0.3 dB, the addition of the stabilization circuits stabilizes the amplifier’s operation throughout the frequency band.

In circuits where low noise and high gain are important, a multi-stage structure cascaded with minimum-noise-focused stages and gain-focused stages is typically employed. When evaluating the stability of the multi-stage circuit, it is often concluded that the stability factor K does not guarantee unconditional stability due to its inherent definition. Therefore, a normalized determinant function (NDF) should be used together with the K factor to effectively identify and suppress the potentially unstable cases [32,33,34,35,36].

The NDF is expressed as $\mathrm{NDF}\left(\omega \right)=\frac{\left|Y\left(\omega \right)\right|}{\left|Y_{0}\left(\omega \right)\right|}$, which represents the ratio of the admittance matrix |Y(ω)| at the suspected node (in this case, the gate and drain nodes of the transistor) to the admittance matrix $\left|Y_{0}\left(\omega \right)\right|$, where all active elements in the circuit are passively transformed. A suspected node refers to a node where oscillation is likely to occur, and the passive transformation of an active element is achieved by setting its transconductance to a non-positive value. The overall NDF for the circuit is determined by multiplying the individual NDF values calculated at each suspected node. The calculation of the NDF and its encirclement around the origin of the NDF polar plot is performed in the ADS circuit simulator from Keysight, where an encirclement value of 1 indicates the threshold for instability in the circuit.

In the polar plot of the NDF versus frequency, more than one complete clockwise rotation around the origin indicates the presence of poles in the right-half plane (RHP) of the circuit’s transfer function, hinting at instability. In the NDF polar plot, it is generally not easy to visually identify whether the NDF trace completes a full rotation or more. Therefore, an encirclement function is used to numerically count how many times the NDF trace rotates from the origin on the polar chart.

**Figure 4:**
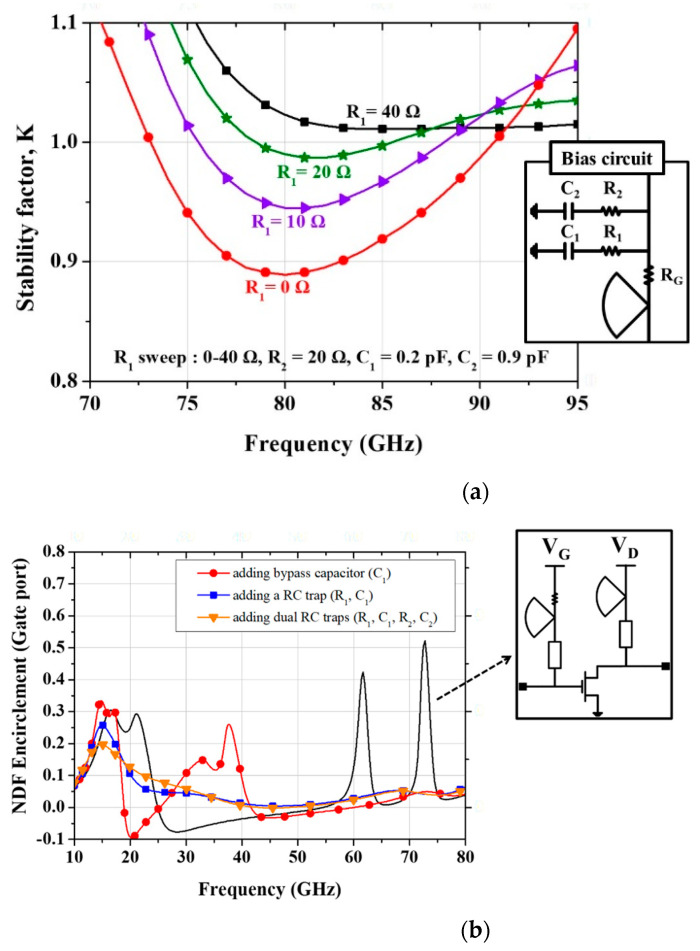
Effects of RC stabilization traps in the bias networks: (**a**) Variation in the stability factor K with respect to the change in the resistor value (R_1_) of the RC stabilization traps; (**b**) Change in NDF encirclement waveforms depending on whether the RC traps are inserted.

Figure 4 shows the effects of RC stabilization traps in the bias networks on the stability factor and NDF encirclement. Figure 4a shows the variation in the stability factor K as R_1_ in the RC trap changes from 0 to 40 Ω. It is observed that the low-frequency stability factors improve as the value of R_1_ increases. Figure 4b presents the NDF encirclement waveforms with and without the RC traps. In all cases, the encirclement remains below 1 across the entire frequency band, indicating the circuit’s stable operation. However, in certain cases, the NDF value shows peaks and approaches at specific frequencies, posing a risk of instability. When the bias networks include only radial stubs without RC traps or bypass capacitors, the risk of instability increases around 60 GHz and 70 GHz. The inclusion of a bypass capacitor C_1_ reduces the oscillation risk around 60 GHz and 70 GHz, but increases it in the 30–40 GHz range. Adding R_1_ in series with the bypass capacitor C_1_ to form a single RC trap in the bias network slightly reduces the instability risk in the 15–20 GHz range and eliminates it in the 30–40 GHz range. Furthermore, the addition of a second RC trap further reduces the risk of residual oscillation in the 15–20 GHz range.

**Figure 5:**
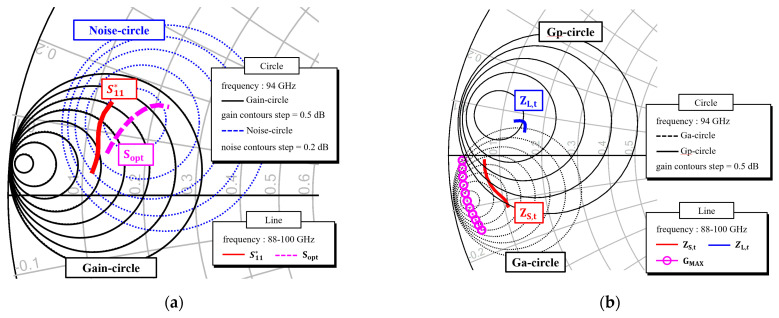
Optimum impedance traces of the transistor stabilized with dual RC traps: (**a**) Gain and noise circles and optimum impedance traces for the minimum noise figure of the 4 × 15 μm transistor; (**b**) Gain circles and optimum impedance traces for the maximum gain of the 4 × 25 μm transistor.

Figure 5a shows the gain and noise circles at 94 GHz, together with the conjugated input reflection coefficient S_11_* and the optimum noise source impedance S_opt_ in the 88–100 GHz range for the 4 × 15 µm transistor stabilized with dual RC traps, as depicted in Figure 3. As the voltage standing wave ratio (VSWR) between S_11_* and S_opt_ is approximately 2, the trade-off impedance trace for the noise figure and input return loss can be determined and its matching circuit can be easily implemented with distributed microstrip elements.

The available gain input circles (Ga-circle) represent the locus of source impedances for a given gain below the maximum available gain, while the power gain output circles (Gp-circle) represent the locus of load impedances for a given gain below the maximum available gain [37,38]. Figure 5b illustrates the Ga-circles and Gp-circles at 94 GHz of the 4 × 25 µm transistor with the stabilization RC traps, as shown in Figure 3. The source and load impedances for the maximum gain have relatively low values, making the impedance matching challenging. As a result, some loss is tolerated in order to obtain the targeted impedances (Z_S,t_ and Z_L,t_), as shown in Figure 5b in the 88–100 GHz range.

### 2.3. Circuit Design and Stability Evaluation

**Figure 6:**
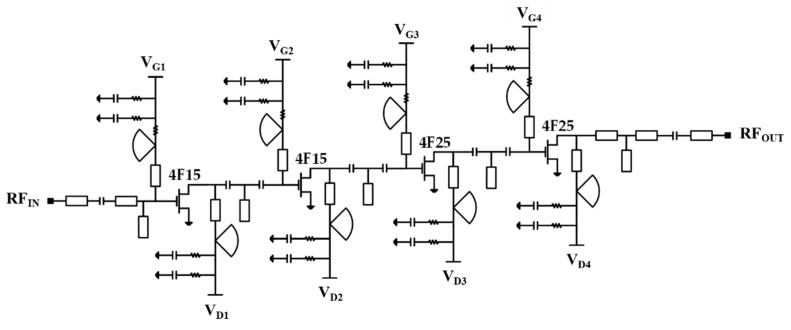
Schematic circuit diagram of the W-band low-noise amplifier MMIC.

Figure 6 presents the complete circuit diagram of the designed four-stage low-noise amplifier MMIC. The LNA is configured in a four-stage cascaded amplifier topology, where the first two stages are optimized for the minimum noise figure, and the last two stages are conjugately matched to the input and output impedances of the transistors for high gain. The distributed passive elements in matching circuits are designed using a 2.5-dimensional electromagnetic momentum simulation in the ADS simulator, and are co-simulated with the transistor nonlinear models provided in the process design kit (PDK). To simultaneously achieve DC blocking and impedance matching, small-sized Metal–Insulator–Metal (MIM) capacitors are utilized, whose self-resonance frequencies (SRFs) are higher than the designed frequency band.

**Figure 7:**
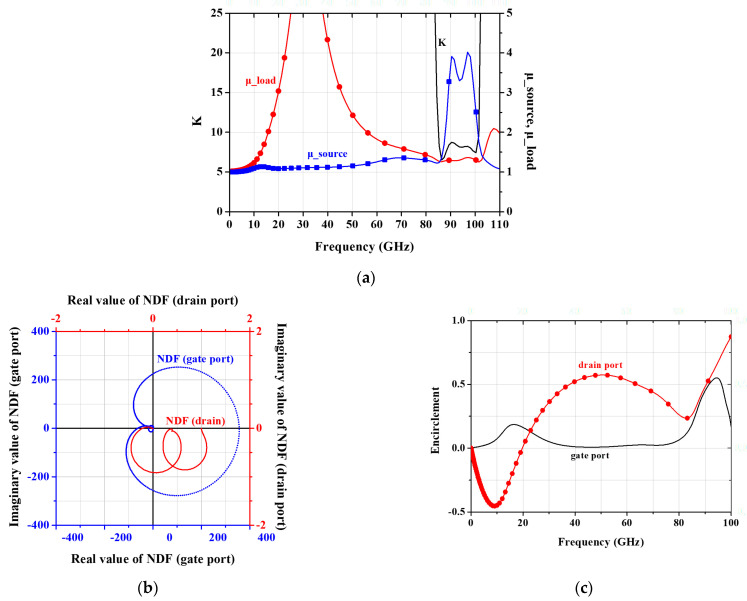
Stability analysis of the designed low-noise amplifier MMIC: (**a**) Stability factors K and μ at the source and load sides; (**b**) NDF polar plot; (**c**) NDF encirclement.

Figure 7 depicts the stability analysis of the designed low-noise amplifier. The analysis includes commonly used stability factors and the network determinant functions at the gate and drain nodes across the full frequency range. The stability evaluation is performed at all suspected nodes, primarily the gate and drain nodes of the transistors in the circuit. The results confirm that the stability factors K and μ consistently exceed 1 throughout the entire frequency range. Additionally, the NDF traces satisfy the Nyquist criterion, with no complete encirclement around the origin, confirming the circuit’s stability.

## 3. Measurement

**Figure 8:**
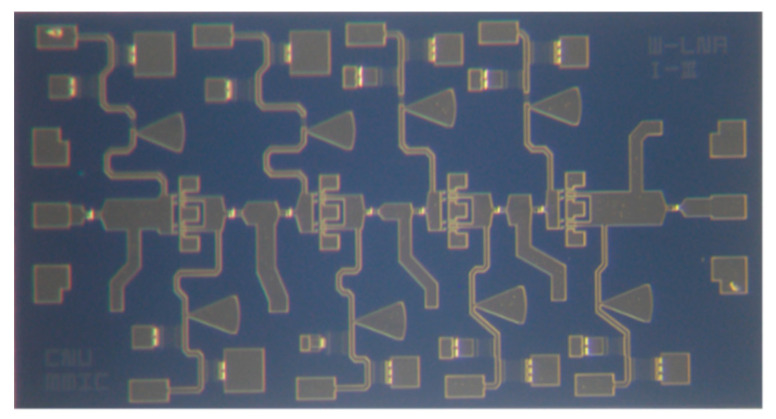
Photograph of the fabricated W-band low-noise amplifier MMIC (area: 1 × 2 mm^2^).

Figure 8 shows the fabricated low-noise amplifier, which occupies an area of 1 × 2 mm^2^. The MMIC chip is mounted on the bottom plate of the metal package using epoxy bonding, and single-layer capacitors (SLCs) with values of 10 nF and 100 pF are placed adjacent to the chip to make bypassing shunt paths for low-frequency signals. The performance evaluation is performed through S-parameter and noise figure measurements using an on-wafer probe system, a vector network analyzer (Keysight N5234B, Keysight, Santa Rosa, CA, USA), WR10 frequency extender modules (Keysight WR10-VNAX), and a mm wave test controller (Keysight N5295A).

**Figure 9:**
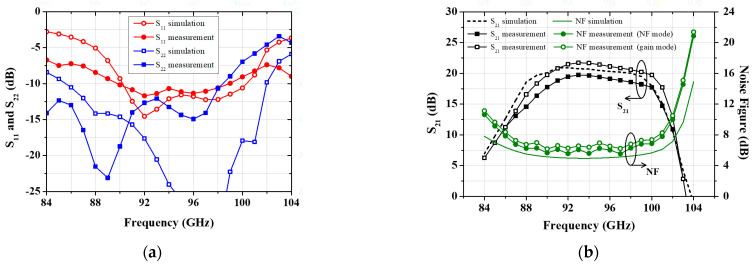
Measured S-parameter results and noise figures of the W-band low-noise amplifier: (**a**) Input and output return loss; (**b**) Gain and noise figure.

The S-parameter measurement is performed under two bias conditions, one for low noise and the other for high gain. For the low-noise mode, all transistors are biased under class AB conditions, and for the high-gain mode, the last two transistors are set to the bias conditions allowing a higher drain current. As shown in Figure 9, the simulation results of the four-stage LNA MMIC show a linear gain of 20–20.1 dB and a noise figure of 4.95–5.23 dB over the 90–98 GHz frequency range, with the input and output return loss better than 10 dB. Figure 9 compares the measurement results of the low-noise amplifier MMIC with the simulation results under the bias conditions of V_DS_ = 2 V, I_DS1,2_ = 18 mA, and I_DS3,4_ = 30 mA. I_DS1,2_ represents the drain current for the first two stages, while I_DS3,4_ corresponds to the drain current for the last two stages in the low-noise mode. In the low-noise mode, the noise figure is measured to be 5.6–6.2 dB with an associated gain of 17.8–19.8 dB. For high-gain operation, the bias conditions of the final two stages are increased to V_DS_ = 2 V, I_DS1,2_ = 18 mA, and I_DS3,4_ = 44 mA, enhancing the gain with a minimal impact on the noise figure. Under the high-gain bias conditions, the LNA achieves a gain of 19.8–21.7 dB and a noise figure of 6.2–6.9 dB. The comparative performance improvement between the two modes is shown in Figure 9b.

Table 1 compares the results of this work with those of previously published W-band LNAs [16,17,18,39,40,41,42]. The fabricated W-band LNA MMIC demonstrates a better gain performance than [17,18], and shows a similar gain performance and lower DC power consumption compared to [40,41]. Although it has a slightly higher noise figure, compared to [42], our work is five times smaller in size while achieving a similar gain performance. The fabricated LNA MMIC demonstrates that the gain performance is comparable to other state-of-the-art design results, while maintaining an area-efficient performance and consuming relatively little DC power, thereby highlighting its competitiveness.

## 4. Conclusions

This work presents a W-band GaAs pHEMT low-noise amplifier MMIC with improved stability over the 90–98 GHz frequency range, which is designed and fabricated using a 0.1 μm GaAs pHEMT process. The primary goal of this work is to mitigate potential instabilities and ensure stable circuit operation through the proposed dual RC traps in the bias networks. The element values for the dual RC traps for enhancing the circuit’s stability over the entire frequency range were determined through a rigorous stability analysis, which was performed using conventional stability factors, K and μ, and network determinant functions based on admittance matrices. The four-stage LNA MMIC fabricated with the proposed dual RC traps achieves a linear gain of 17.8–19.8 dB and a noise figure of 5.6–6.2 dB over the 90–98 GHz frequency range, while consuming a low DC power of 96 mW. The developed W-band LNA MMIC demonstrates a good balance between the gain and noise figure while maintaining excellent stability. These characteristics make the proposed LNA MMIC a good candidate for W-band transceiver modules, which require low power consumption, high gain, a low noise figure, and a small size.

## Figures and Tables

**Table 1:** Comparison of the results from this work with those of the previously published W-band low-noise amplifier MMICs using a 0.1 μm GaAs process.

Work	Frequency (GHz)		Gain (dB)	Noise Figure (dB)	DC Power (mW)	Size (mm^2^)
[16]	71–86		20.5–23	2.7–4.3	262.5	3.9 mm^2^
[17]	80–94		≥11	5 ^†^	25.5	1.4 mm^2^
[18]	94–105		10–12.5	4.7–5	123	1.7 mm^2^
[39]	83–96		20.5 ^†^	5.3–6.5	108	2.1 mm^2^ **
[40]	75–110		17–22	3.5–4.5	140	1.1 mm^2^
[41]	75–110		20 ^†^	5.5 *	138	1.2 mm^2^
[42]	92–115		19 ^†^	4.6 ^†^	80	10 mm^2^
This work	90–98	Low-noise mode	17.8–19.8	5.6–6.2	96	2 mm^2^
		High-gain mode	19.8–21.7	6.2–6.9	124	

(*): simulated performance. (**): estimated value. (†): average value.

## Data Availability

The original contributions presented in this study are included in the article. Further inquiries can be directed to the corresponding author.
